# Supplementary material for: Heat adaptation of phage T7 under an extended genetic code
Source: Virus Evol. 2021 Dec 1;7(2):veab100. doi: 10.1093/ve/veab100 (PMC8923235; doi:10.1093/ve/veab100)
Supplement: veab100_Supp [file veab100_supp.zip › Sequencing_Analysis.zip!/Sequencing_Analysis/A3/evidence/SNP_11.html]

BRESEQ :: Evidence


| Predicted mutation | | | | | | |
| --- | --- | --- | --- | --- | --- | --- |
| evidence | seq id | position | mutation | annotation | gene | description |
| RA | NC\_001604.1 | 27,932 | T→C | intergenic (–/–) | – / – | –/– |

| Read alignment evidence... | | | | | | | | | | | |
| --- | --- | --- | --- | --- | --- | --- | --- | --- | --- | --- | --- |
|  | seq id | position | | ref | new | freq | score (cons/poly) | reads | annotation | genes | product |
| \* | NC\_001604.1 | 27,932 | 0 | T | C | 98.2% | 5478.8 / 3.8 | 1827 | intergenic (–/–) | *–/–* | –/– |
| Reads supporting (aligned to +/- strand):  **ref** base T (3/6);  **major** base C (832/950);  **minor** base A (9/23);  **total** (845/982) | | | | | | | | | | | |
| Fisher's exact test for biased strand distribution *p*-value = 4.78e-02 | | | | | | | | | | | |
| Kolmogorov-Smirnov test that lower quality scores support variant *p*-value = 3.18e-01 | | | | | | | | | | | |

Only 100 of 1828 total aligned reads displayed.  

`TTCCTCAAGACTATCGGTGCGGTATTCCATGAAGAGTACACACGAGATGGTCAATTTCAGTTATTTACAATCACGAAAGGAGGATAACCATATGTGTTGGGCAGCCGCAATACCTATCGCTATATCTGGCGCTCAGGCTATCAGTGGTCAGAACGCTCAGGCCAAAATGATTGCCGCTCAGACCGCTGCTGGTCGTCGTCAAGCTATGGAAATCATGAGGCAGACGAACATCCAGAATGCTGACCTATCGTTGCAAGCTCGAAGTAAACTTGAGGAAGCGTCCGCCGAGTTGACCTCACAGAACATGCAGAAGGTCCAAGCTATTGGGTCTATCCGAGCGGCTATCGGAGAGAGTATGCTTGAAGGTTCCTCAATGGACCGCATTAAGCGAGTCACAGAAGGACAGTTCATTCGGGAAGCCAATATGGTAACTGAGAACTATCGCCGTGACTACCAAGCAATCTTCGCACAGCAACTTGGTGGTACTCAAAGTGCTGCAAGTCAGATTGACGAAATCTATAAGAGCGAACAGAAACAGAAGAGTAAGCTACAGATGGTTCTGGACCCACTGGCTATCATGGGGTCTTCCGCTG  >  NC_001604.1/27637‑28229`  

                                                                                                                                                                                                                                                                                                       `|`                                                                                                                                                                                                                                                                                                           
`acccTCAAGACTATCGGTGCGGTATTCCATGAAGAGTACACACGATATGGTCAATTTCAGTTATTTACAATCACGAAAGGAGGATAACCATATGTGTTGGGCAGCCGCAATACCTATCGCTATATCTGGCGCTCAGGCTATCAGTGATCAGAACGCTCAGGCCAAAATGATTGCCGCTCAGACCGCTGCTGGTCGTCGTCAAGCTATGGAAATCATGAGGCAGACGAACATCCAGAATGCTGACCTATCGTTGCAAGCTCGAAGTAAACTTGAGGAAGCGTCCGCCGAGTTGACCCCACAg                                                                                                                                                                                                                                                                                                      <  1:240811/299‑1 (MQ=255)`  
 `tCCTCAAGACTATCTGTGCGGTATTCCATGAAGAGTACACACGAGATTGTCAATTTCCGTTATTTACAATCACGAAAGGAGTATAACCATATGTGTTGTGCAGCCGCAATACCTATCGCTATATCTGGCGCTCAGGCTATCAGTGATCAGAACGCTCAGTCCAAAATGATTGCCGCTCAGACCGCTGCTGGTCGTCGTCAAGCTATTGAAATCATGAGGCAGAAGAACATCCAGAATGCTGACCTATCGTTGCAAGCTCGAAGTAAACTTGAGGAAGCGTCCGCCGCGTTGACCCCACAGa                                                                                                                                                                                                                                                                                                     <  1:47271/301‑1 (MQ=255)`  
   `gtataagagacaggTGCGGTATTCCATGAAGAGTACACACGAGATGGTCAATTTCAGTTATTTACAATCACGAAAGGAGGATAACCATATGTGTTGGGCAGCCGCAATACCTATCGCTATATCTGGCGCTCAGGCTATCAGTGATCAGAACGCTCAGGCCAAAATGATTGCCGCTCAGACCGCTGCTCGTCGTCGTCAAGCTATGGAAATCATGAGGCAGACGAACATCCAGAATGCTGACCTATCGTTGCAAGCTCGAAGTAAACTTGAGGAAGCGTCCGCCGAGTTGACCCCACAGAAc                                                                                                                                                                                                                                                                                                   <  1:38393/289‑1 (MQ=255)`  
     `gagatgtgtataagagacaggccATGAAGAGGAAAAACGAGATGGTCAATTGCAGTTATTTACAATCACGAAAGGAGGATAACCATATGTGTTGGGCAGCCGCAATACCTATCGATATATCTGGCGCTCAGGCTATCAGTGATCAGAACGCTCAGGCCAAAATGATTGCCGCGCAGAACGCTGCTGGTCGTCATCAAGCTATGGAAATCATGAGGCAGACGAACATCCAGAATGCTGACCTATCGTTGCAAGCTCGAAGTAAACTTGAGGAAGCGTCCGCCGAGTTGACCCCACAGAACAt                                                                                                                                                                                                                                                                                                 <  1:110448/280‑1 (MQ=255)`  
     `cAAGACTATCGGTGCGGTATTCCATGAAGAGTACACACGAGATGGTCAATTTCAGTTATTTACAATCACGAAAGGAGGATAACCATATGTGTTGGGCAGCCGCAATACCTATCGCTATATCTGGCGCTCAGGCTATCAGTGATCAGAACGCTCAGGCCAAAATGATTGCCGCTCAGACCGCTGCTGGTCGTCGTCAAGCTATGGAAATCATGAGGCAGACGAACATCCAGAATGCTGACCTATCGTTGCAAGCTCGAAGTAAACTTGAGGAAGCGTCCACCGAGTTGACACCACAGAACAt                                                                                                                                                                                                                                                                                                 >  1:270820/1‑301 (MQ=255)`  
          `cTATCGGTGCGGTATTCCATGAAGAGTACACACGAGATGGTCAATTTCAGTTATTTACAATCACGAAAGGAGGATAACCATATGTGTTGGGCAGCCGCAATACCTATCGCTATATCTGGCGCTCAGGCTATCAGTGATCAGAACGCTCAGGCCAAAATGATTGCCGCTCAGACCGCTGCTCGTCGTCGTCAAGCTATGGAAATCATGAGGCAGACGAACATCCAGAATGCTGACCTATCGTTGCAAGCTCGAAGTAAACTTGAGGAAGCGTCCGCCGAGTTGACCCAACAGAActgtctct                                                                                                                                                                                                                                                                                            >  1:297788/1‑294 (MQ=255)`  
           `aacaggTGCGGTATTCCATGAAGAGTACACACGAGATGGTCAATTTCAGTTATTTACAATCACGAAAGGAGGATAACCATATGTGTTGGGCAGCCGAAATACCTATCGCTATATCTGGCGCTAAGGCTATCAGTGATCAGAACGCTCAGGCCAAAATGATTGCCGCTCAGACCGCTGCTCGTCGTCGTCAAGCTATGGAAATCATGAGGCAGACGAACATCCAGAATGCTGACCTATCGTTGCAAGCTCGAAGTAAACTTGAGGAAGCGTCCGCCGAGTTGACCCCACAGAACATGCAGaa                                                                                                                                                                                                                                                                                           <  1:136015/297‑1 (MQ=255)`  
                     `gTATTCCATGAAGAGTACACACGAGATGGTCAATTTCAGTTATTTACAATCACGAAAGGAGGATAACCATATGTGTTGGGCAGCCTCAATACCTATCGCTATATCTGGCGCTCAGGCTATCAGTGATCAGAACGCTCAGGCCAAAATGATTGCCGCTCAGACCGCTGCTAGTCGTCGTCAAGCTATGGAAATCATGAGGCAGACGAACATCCAGAATGCTGACCTATCGTTGCAAGCTCGAAGTAAACTTGAGGAAGCGTCCGCCGAGTTGACCCCACAGAACactgtctcttatacacat                                                                                                                                                                                                                                                                                 >  1:105157/1‑284 (MQ=255)`  
                     `gTATTCCATGAAGAGTACACACGAGATGGTCAATTTCAGTTATTTACAATCACGAAAGGAGGATAACCATATGTGTTGGGCAGCCGCAATACCTATTGCTATATCTGGCGCTCAGGCTATCAGTGATCAGAACGCTCAGGCCAAAATGATTGCCGCTCAGACCGCTGCTGGTCGTCGTCAAGCTATGGAAATCATGAGGCAGACGAACATCCAGAATGCTGACCTATCGTTGCAAGCTCGAAGTAAACTTGAGGAAGCGTCCGCCGAGTTGACCCCACAGAACATGCAGAAGGTCCAAGCt                                                                                                                                                                                                                                                                                 >  1:85325/1‑301 (MQ=255)`  
                     `gTATTCCATGAAGAGTACACACGAGATGGTCAATTTCAGTTATTTACAATCACGAAAGGAGGATAACCATATGTGTTGGGCAGCCGCAATACCTATCGCTATATCTGGCGCTCAGGCTATCAGTGATCAGAACGCTCAGGCCAAAATGATTGCCGCTCAGACCGCTGCTGGTCGTCGTCAAGCTATGGAAATCATGAGGCAGACGAACATCCAGAATGCTGACCTATCGTTGCAAGCTCGAAGTAAACTTGAGGAAGCGTCCGCCGAGTTGACCCCACAGAACATGCAGAAGGTCCAAGCt                                                                                                                                                                                                                                                                                 <  1:313227/301‑1 (MQ=255)`  
                          `ccATGAAGAGTACACACGAGATGGTCAATTTCAGTTATTTACAATCACGAAAGGAGGATCACCATATGTGTTGGGCAGCCGCAATACCTATCGCTATATCTGGCGCTCAGGCTCTCAGTGATCAGAACGCTCAGGCCAAAATGATTGCCGCTAAGACCGCTGCTGGTCGTCGTCAAGCTATGGAAATCATGAGGCAGACGAACATCCAGAATGCTGACCTATCGTTGCAAGCTCGAAGTAAACTTGAGGAAGCGTCCGCCGAGTTGACCCCACATACCATGCAGAAGGTCCAAGCTATTgg                                                                                                                                                                                                                                                                            >  1:181423/1‑301 (MQ=255)`  
                          `ccATGAAGAGTACACACGAGATGGTCAATTTCAGTTATTTACAATCACGAAAGGAGGATAACCATATGTGTTGGGCAGCCGCAATACCTATCGCTATATCCGGCGCTCAGGCTATCAGTGATCAGAACGCTCAGGCCAAAATGATTGCCGCTCAGACCGCTGCTGGTCGTCGTCAAGCTATGGAAATCATGAGGCAGACGAACATCCAGAATGCTGACCTATCGTTGCAAGCTCGAAGT‑AACTTGAGGAAGCGTCCGCCGAGTTGACCCCACAGAACCTgtctcttatacacatctccgac                                                                                                                                                                                                                                                                           >  1:150640/1‑280 (MQ=255)`  
                           `cATGAAGAGTACACACGAGATGGTCAATTTCAGTTATTTACAATCACGAAAGGAGGATAACCATATGTGTTGGGCAGCCGCAATACCTATCGCTATATCTGGCGCTCAGGCTATCAGTGATCAGAACGCTCAGGCCAAAATGATTGCCGCTCAGACCGCTGCTGGTCGTCGTCAAGCTATGGAAATCATGAGGCAGACGAACATCCAGAATGCTGACCTATCGTTGCAAGCTCGAAGTAAACTTGAGGAAGCGTCCGCCGAGTTGACCCCACAGAACATGCAGAAGGTCCAAGCTATTggg                                                                                                                                                                                                                                                                           <  1:175078/301‑1 (MQ=255)`  
                               `aaGAGTACACACGAGATGGTCAATTTCAGTTATTTACAATCACGAAAGGAGGATAACCATATGTGTTGGGCAGCCGCAATACCTATCGCTATATCTGGCGCTCAGGCTATCAGTGATTAGAACGCTCAGGCCAAAATGATTGCCGCTCAGACCGCTGCTCGTCGTCGTCAAGCTATGGAAATCATGAGGCAGACGAACATCCAGAATGCTGACCTATCGTTGCAAGCTCGAAGTAAACTTGAGGAAGCGTCCGCCGAGTTGACCCCACAGAACATGCAGAAGGTCCAAGCTATTGGGTCtt                                                                                                                                                                                                                                                                       >  1:171924/1‑300 (MQ=255)`  
                                 `gagTACACACGAGATGGTCAATTTCAGTTATTTACAATCACGAAAGGAGGATAACCATATGTGTTGGGCAGCCGCAATACCTATCGCTATATCTGGCGCTCAGGCTATCAGTGATCAGAACGCTCAGGCCAAAATGATTGCCGCTCAGACCGCTGCTGGTCGTCGTCAAGCTATGGAAATCATGAGGCAGACGAACATCCAGAATGCTGACCTATCGTTGCAAGCTCGAAGTAAACTTGAGGAAGCGTCCGCCGAGTTGACCCCACAGAACATGCAGAAGGTCCAAGCTATTGGGTCTATc                                                                                                                                                                                                                                                                     >  1:59879/1‑301 (MQ=255)`  
                                      `cacaaGGGATTGTCAATTTCAGTTATTTACAATCACGAAAGGAGGATAACCATATGTGTTGGGCAGCCGCAATACCTATCGCTATATCTGGCGCTCAGGCTATCAGTGATCAGAACGCTCAGGCCAAAATGATTGCCGCTCAGACCGCTGCTGGTCGTCGTCAAGCTATGGAAATCATGAGGCAGACGAACATCCAGAATGCTGACCTATCGTTGCAAGCTCGAAGTAAACTTGAGGAAGCGTTCGCCGAGTTGACCCCACAGAACATGCAGAAGGTCCAAGCTATTGGGTCTATCCGAGc                                                                                                                                                                                                                                                                <  1:310450/301‑1 (MQ=255)`  
                                            `agaTGGTCAATTTCAGTTATTTACAATCACGAAAGGAGGATAACCATATGTGTTGGGCAGCCGCAATACCTATCGCTATATCTGGCGCTCAGGCTATCAGTGATCAGAACGCTCAGGCCAAAATGATTGCCGCTCAGACCGCTGCTGGTCGTCGTCAAGCTATGGAAATCATGAGGCAGACGAACATCCAGAATGCTGACCTATCGTTGCAAGCTCGAAGTAAACTTGAGGAAGCGTCCGCCGAGTTGACCCCACAGAACATGCAGAAGGTCCAAGCTATTGGGTCTATCCGAGCGGCTAt                                                                                                                                                                                                                                                          >  1:322369/1‑301 (MQ=255)`  
                                                 `gTCAATTTCAGTTATTTACAATCCCGAAAGGAGGATAACCATACGTGTTGGGCAGCCGCAATACCTATCGCTATATCTGGCTCTCAGGCTATCAGTGATCAGAACGCTCAGGCCAAAATGATTGCCGCTCAGACCGCTGCTCGTCGTCGTCAAGCTATGGAAATCATGAGGCAGACGAACATCCAGAATGCTGACCTATCGTTGCAAGATCGAAGTAAACTTGAGGAAGCGTCCGCCGAGTTGACCCCACAGAACATGCAGAAGGTCCAAGCTATTGGGTCTATCCGAGCGGCTATCGgag                                                                                                                                                                                                                                                     <  1:36209/301‑1 (MQ=255)`  
                                                     `aTTTCAGTTATTTACAATCACGAAAGGAGGATAACCATATGTGTTGGGCAGCCGCAATACCTATCGCTATATCTGGCGCTCAGGCTATCAGTGATCAGAACGCTCAGGCCAAAATGATTGCCGCTCAGACCGCTGCTGGTCGTCGTCAAGCTATGGAAATCATGAGGCAGACGAACATCCAGAATGCTGACCTATCGTTGCAAGCTCGAAGTAAACTTGAGGAAGCGTCCGCCGAGTTGACCCCACAGAACATGCAGAAGGTCCAAGCTATTGGGTCTATCCGAGCGGCTATCGGagagag                                                                                                                                                                                                                                                 <  1:143066/301‑1 (MQ=255)`  
                                                         `cAGTTATTTACAATCACGAAAGGAGGATAACCATATGTGTTGGGCAGCCGCAATACCTATCGCTATATCTGGCGCTCAGGCTATCAGTGATCAGAACGCTCAGGCCAAAATGATTGCCGCTCAGACCGCTGCTGGTCGTCGTCAAGCTATGGAAATCATGAGGCAGACGAACATCCAGAATGCTGACCTATCGTTGCAAGCTCGAAGTAAACTTGAGGAAGCGTCCGCCTAGTTGACCCCACAGAACATGCAGAAGGTCCAAGCTATTGGGTCTATCCGAGCGGCTATCGGAGAGAGTATg                                                                                                                                                                                                                                             >  1:222928/1‑301 (MQ=255)`  
                                                           `gTTATTTACAATCACGAAAGGAGGATAACCATATGTGTTGGGCAGCCGCAATACCTATCGCTATATCTGGCGCTCAGGCTATCAGTGATCAGAACGCTCAGGCCAAAATGATTGCCGCTCAGACCGCTGCTCGTCGTCGTCAAGCTATGGAAATCATTAGGCAGACGAACATCCAGAATGCTGACCTATCGTTTCAAGCTCGAAGTAAACTTGAGAAAGCGTCCGCCGAGTTGACCCCACAGAACATGCAGAAGTTCCAAGCTATTGGGTCTATCCGAGCGGCTATCGGAGAGAGTATGCt                                                                                                                                                                                                                                           >  1:147449/1‑301 (MQ=255)`  
                                                           `gTTATGTACAATCACGAAAGGAGGATAACCATATGTGTTGGGCAGCCGCAATACCTATCGCTATATCTGGCGCTCAGGCTATCAGTGATCAGAACGCTCAGGCCAAAATGATTGCCGCTCAGACCGCTGCTCGTCGTCGTCAAGCTATGGAAATCATGAGGCAGACGAACATCCAGAATGCTGACCTATCGTTGCAAGCTCGAAGTAAACTTGAGGAAGCGTCCGCCGAGTTGACCCCACAGAACATGCAGAAGGTCCAAGCTATTGGGTCTAACCGAGCGGCTATCGGAGAGAGTATGCt                                                                                                                                                                                                                                           <  1:251198/301‑1 (MQ=255)`  
                                                           `attATTTACAATCACGAAAGGAGGATAACCAGATGTGTTGGGCAGCCGCGATACCTATCGCTATATCTGGCGCTCAGGCTATCAGTGATCAGAACGCTCAGGCCAAAATGATTGCCGCTCAGACCGCTGCTGGTCGTCGTCAAGCTATGGAAATCATGAGGCAGACGAACATCCAGAATGCTGACCTATCGTTGCAAGCTCGAAGTAAACTTGAGGAAGCGTCCGCCGAGTTGACCCCACAGAACATGCAGAAGGTCCAAGCTATTGGGTCTATCCGAGCGGCTATCGGAGAGAGTATGCt                                                                                                                                                                                                                                           <  1:61928/300‑1 (MQ=255)`  
                                                                  `aCAATCACGAAAGGAGGATAACCATAGGTGGTTGGCAGCCGCAATACCTATCGCTATATCTGGCGCTCAGGCTATCAGTGATCAGAACGCTCAGGCCAAAATGATTGCCGCTCAGACCGCTGCTGGTCGTCGTCAAGCTATGGAAATCATGAGGCAGACGAACATCCAGAATGCTGACCTATCGTTGCAAGCTCGAAGTAAACTTGAGGAAGCGTCCGCCGAGTTGACCCCACAGAACATGCAGAAGGTCCAAGCTATTGGGTCTATCCGAGCGGCTATCGGAGAGAGTATGCTTGAAGGt                                                                                                                                                                                                                                    <  1:219877/301‑1 (MQ=255)`  
                                                                       `cACGAAAGGAGGATAACCATATGTGTTGGGCAGCCGCAATACCTATCGCTATATCTGGCGCTCAGGCTATCAGTGATCAGAACGCTCAGGCCAAAATGATTGCCGCTCAGACCGCTGCTCGTCGTCGTCAAGCTATGGAAATCATGAGGCAGACTAACATCCAGAATGCTGACCTATCGTTGCAAGCTCGAAGTAAACTTGAGGAAGCGTCCGCCGAGTTGACCCCACAGAACATGCAGAAGGTCCAAGCTATTGGGTCTATCCGAGCGGCTATCGGAGAGAGTATGCCTgtctcttatac                                                                                                                                                                                                                               >  1:191167/1‑291 (MQ=255)`  
                                                                       `cACGAAAGGAGGATAACCATATGTGTTGGGCAGCCGCAATACCTATCGCTATATCTGGCGCTCAGGCTATCAGTGATCAGAACGCTCAGGCCAAAATGATTGCCGCTCAGACCGCTGCTCGTCGTCGTCAAGCTATGGAAATCATGAGGCAGACGAACATCCAGAATGCTGACCTATCGTTGCAAGCTCGAAGTAAACTTGAGGAAGCGTCCGCCGAGTTGACCCCACAGAACATGCAGAAGGTCCAAGCTATTGGGTCTATCCGAGCGGCTATCGGAGAGAGTATGCTTGAAGGTTCCTc                                                                                                                                                                                                                               >  1:65971/1‑301 (MQ=255)`  
                                                                       `cACGAAAGGAGGATAACCATATGGGTTGGGCAGCCGCAATACCTATCGCTATATCTGGCGCTAAGGCTATCAGTGATCAGAACGCTCAGGCCAAAATGATTGCCGCTCAGACCGCTGCTCGTCGTCGTCAAGCTATGGAAATCATGAGGCAGACGAACATCCAGAATGCTGACATATCGTTGCAAGCTCGAAGTAAACTTGAGGAAGCGTCCGCCGAGTTGACCCCACAGAACATGCAGAAGGTCCAAGCTATTGGGTCTATCCGAGCGGCTATCGGAGAGAGTATGCTTGAAGGTTCCTc                                                                                                                                                                                                                               <  1:44157/301‑1 (MQ=255)`  
                                                                           `aaaGGAGGATAACCATATGTGTTGGGCAGCCGCAATACCTATCGCTATATCTGGCGCTCAGGCTATCAGTGATCAGAACGCTCAGGCCAAAATGATTGCCGCTCAGACCGCTGCTGGTCGTCGTCAAGCTATGGAAATCATGAGGCAGACGAACATCCAGAATGCTGACCTATCGTTGCAAGCTCGAAGTAAACTTGAGGAAGCGTCCGCCGAGTTGACCCCACAGAACATGCAGAAGGTCCAAGCTATTGGGTCTATCCGAGCGGCTATCGGAGAGAGTATGCTTGAAGGTTCCTCAATg                                                                                                                                                                                                                           >  1:75749/1‑301 (MQ=255)`  
                                                                              `ggaggaTAACCATATGTGTTGGGCAGCCGCAATACCTATCGCTATATCTGGCGCTCAGACTATCAGTGATCAGAACGCTCAGGCCAAAATGATTGCCGCTCAGACCGCTGCTCGTCGTCGTCAAGCTATGGAAATCATGAGGCAGACGAACATCCAGAATGCTGACCTATCGTTGCAAGCTCGAAGTAAACTTGAGGAAGCGTCCGCCGAGTTGACCCCACAGAACATGCAGAAGGTCCAAGCTATTGGGTCTATCCGAGCGGCTATCGGAGAGAGTATGCTTGAAGGTTCCTCAATGGAc                                                                                                                                                                                                                        >  1:279078/1‑301 (MQ=255)`  
                                                                              `ggaggaTAACCATATGTGTTGGGAAGCCGCAATACCTATCGCTATATCTGGCGCTCAGGCTATCAGTGATCAGAACGCTCAGGCCAAAATGATTGCCGCTCAGACCGCTGCTCGTCGTCGTCAAGCTATGGAAATCATGAGGCAGACGAACATCCAGAATGCTGACCTATCGTTGCAAGCTCGAAGTAAACTTGAGGAAGCGTCCGCCGAGTTGACCCCACAGAACATGCAGAAGGTCCAAGCTATTGGGTCTATCCGAGCGGCTCTCGGAGAGAGTATGCTTGAAGGTTCCTCAATGGAc                                                                                                                                                                                                                        >  1:93183/1‑301 (MQ=255)`  
                                                                                      `aCCATATGTGTTGGGCAGCCGCAATACCTATCGCTATATCTGGCGCTCAGGCTATCAGTGATCAGAACGCTCAGGCCAAAATGATTGCCGCTCAGACCGCTGCTCGTCGTCGTCAAGCTATGGAAATCATGAGGCAGACGAACATCCAGAATGCTGACCTATCGTTGCAAGCTCGAAGTAAACTTGAGGAAGCGTCCGCCGAGTTGACCCCACAGAACATGCAGAAGGTCCAAGCTATTGGGTCTATCCGAGCGGCTATCGGAGAGAGTATGCTTGAAGGTTCCTCAATggctgtctctta                                                                                                                                                                                                                >  1:243663/1‑291 (MQ=255)`  
                                                                                       `ccATATGTGTTGGGCAGCCGCAATACCTATCGCTATATCTGGCGCTCAGGCTATCAGTTATCAGAACGCTCAGGCCACAATGATTGCCGCTCAGACCGCTGCTGGTCGTCGTCAAGCTATGGAAATCATGAGGCAGACGAACATCCAGAATGCTGACCTATCGTTGCAAGCTCGAAGTAAACTTGAGGAAGCGTCCGCCGAGTTGACCCCACAGAACATGCAGAAGGTCCAAGCTATTGGGTCTATCCGAGCGGCTATCGGAGAGAGTATGCTTGAAGGTTCCTCAATGGACCGCATTAAg                                                                                                                                                                                                               >  1:327414/1‑301 (MQ=255)`  
                                                                                       `ccATATGTGTTGGGCAGCCGCAATACCTATCGCTATATCTGGCGCTCAGGCTATCAGTGATCAGAACGCTCAGGCCAAAATGATTGCCGCTCAGACCGCTGCTCGTCGTCGTCAAGCTATGGAAATCATGAGGCAGACGAACATCCAGAATGCTGACCTATCGTTGCAAGCTCGAAGTAAACTTGAGGAAGCGTACGCCGAGTTGACCCCACAGAACATGCAGAAGGTCCAAGCTATTGGGTCTATCAGAGCGGCTATCGGAGAGAGTATGCTTGAAGGTTCCTCAATGGACCGCATTAAg                                                                                                                                                                                                               >  1:258327/1‑301 (MQ=255)`  
                                                                                        `caaatGTGTTGGGCAGCCGCAATACATATCGCGATAGCTGGGGCTCAGTCAATCAGTGATCAGAACGCTCAGGCCAAAATGATTGCCGCTCAGACCGGTGCTGGTCGTCGTCAAGGTATGGAAATAATGAGGCAGACTAAAATCCACAATGCTGAGATATCGTTGCAAGCTCGAAGTAAACTAGAGGAAGCGTCCGCCGAGTTGACCCCACAGAACATGCAGAAGGTCCAAGCTATTGGGTCTATCCGAGCGGCTATCGGAGAGAGTATGCTTGAAGGTTCCTCAATGGACCGCATTAAGc                                                                                                                                                                                                              <  1:235239/298‑1 (MQ=255)`  
                                                                                          `tatGTGTTGGGCAGCCGCAATACCTATCGCTATATCTGGCGATCAGGCTATCAGTGATCAGAACGCTCAGGCCAAAATGATTGCCGCTCAGACCGCTGCTGGTCGTCGTCAAGCTATGGAAATCATGAGGCAGACGAACATCCAGAATGCTGACCTATCGTTGCAAGCTCGAAGTAAACTTGAGGAAGCGTCCGCCGAGTTGACCCCACAGAACATGCAGAAGGTCCAAGCTATTGGGTCTATCCGAGCGGCTATCGGAGAGAGTATGCTTGAAGGTTccctgtctcttatacacatctcc                                                                                                                                                                                                            >  1:113292/1‑280 (MQ=255)`  
                                                                                                       `gagatgtgtataagagacagatCTGGCGCTCAGGCTATCAGTGATCAGAACGCTCAGGCCAAAATGATTGCCGCTCAGACCGCTGCTCGTCGTCGTCAAGCTATGGAAATCATGAGGCAGACGAACATCCAGAATGCTGACCTATCGTTGCAAGCTCGAAGTAAACTTGAGGAAGCGTCCGCCGAGTTGACCTCACAGAACATGCAGAAGGTCCAAGCTATTGGGTCTATCCGAGCGGCTATCGGAGAGAGTATGCTTGAAGGTTCCTCAATGGACCGCATTAAGCGAGTCACAGAAGGAc                                                                                                                                                                                               <  1:286703/281‑1 (MQ=255)`  
                                                                                                       `gagatgtgtataagagacagatCTGGCGCTCAGGCTATCAGTGATCAGAACGCTCAGGCCAAAATGATTGCCGCTCAGACCGCTGCTCGTCGTCGTCAAGCTATGGAAATCATGAGGCAGACGAACATCCAGAATGCTGACCTATCGTTGCAAGCTCGAAGTAAACTTGAGGAAGCGTCCGCCGAGTTGACCCCACAGAACATGCAGAAGGTCCAAGCTATTGGGTCTATCCGAGCGGCTATCGGAGAGAGTATGCTTGAAGGTTCCTCAATGGACCGCATTAAGCGAGTCACAGAAGGAc                                                                                                                                                                                               <  1:187266/281‑1 (MQ=255)`  
                                                                                                       `agagacagaCCTATCGCTATATCTGGCGCTCAGGCTATCAGTGATCAGAACGCTCAGGCCAAAATGATTGCCGCTCAGACCGCTGCTCGTCGTCGTCAAGCTATGGAAATCATGAGGCAGACGAACATCCAGAATGCTGACCTATCGTTGCAAGCTCGAAGTAAACTTGAGGAAGCGTCCGCCGAGTTGACCCCACAGAACATGCAGAAGGTCCAAGCTATTGGGTCTATCCGAGCGGCTATCGGAGAGAGTATGCTTGAAGGTTCCTCAATGGACCGCATTAAGCGAGTCACAGAAGGAc                                                                                                                                                                                               <  1:789/293‑1 (MQ=255)`  
                                                                                                         `cGCAATACCTATCGCTATATCTGGCGCTCAGGCTATCAGTGATCAGAACGCTCAGGCCAAAATGATTGCCGCTCAGACCGCTGCTCGTCGTCGTCAAGCTATGGAAATCATGAGGCAGACGAACATCCAGAATGCTGACCTATCGTTGCAAGCTCGAAGTAAACTTGAGGAAGCGTCCGCCTAGTTTACCCCACAGAACATGCAGAAGGTCCAAGCTATTGGGTCTATCCGAGCGGCTATCGGAGAGAGTATGCTTGAAGGTTCCTCAATGGACCGCATTAAGCGAGTCACAGAAGGACAg                                                                                                                                                                                             <  1:49504/301‑1 (MQ=255)`  
                                                                                                         `ataagagacagtCGCTATATCTGGCGCTCAGGCTATCAGTGATCAGAACGCTCAGGCCAAAATGATTGCCGCTCAGACCGCTGCTGGTCGTCGTCAAGCTATGGAAATCATGAGGCAGACGAACATCCAGAATGCTGACCTATCGTTGCAAGCTCGAAGTAAACTTGAGGAAGCGTCCGCCGAGTTGACCCCACAGAACATGCAGAAGGTCCAAGCTATTGGGTCTATCCGAGCGGCTATCGGAGAGAGTATGCTTGAAGGTTCCTCAATGGACCGCATTAAGCGAGTCACAGAAGGACAg                                                                                                                                                                                             <  1:184353/290‑1 (MQ=255)`  
                                                                                                          `aaagagacagtCGCTATAGCTGGCGCTCAGGCTATCAGTAAACAGAACTGTCAGGCCAAAATGATTTCCGCTCAGACCGCTGGTGGTCGTCGTAAAGCTATGGAAATCATGAGGCAGACGAACATACAAAATGCTGACCTATCGTTGCAAGCTCGAAGTAAACTTGAGGAAGCGTCCGCCGAGTTGACCCCACAGAACATGCAGAAGGTCCAAGCTATTGGGTCTATCCGAGCGGATATCGGAGAGAGTATGCTTGAAGGTTCCTCAATGGACCGCATTAAGCGAGTCACAGAAGGACAGt                                                                                                                                                                                            <  1:100031/291‑1 (MQ=255)`  
                                                                                                               `aCCTATCGCTATATCTGGCGCTCAGGCTATCAGTGATCAGAACGCTCAGGCCAAAATGATTGCCGCTCAGACCGCTGCTCGTCGTCGTCAAGCTATGGAAATCATGAGGCAGACGAACATCCAGAATGCTGACCTATCGTTGCAAGCTCGAAGTAAACTTGAGGAAGCGTCCGCCGAGTTGACCCCACAGAACATGCAGAAGGTCCAAGCTATTGGGTCTATCCGAGCGGCTATCGGAGAGAGTATGCTTGAAGGTTCCTCAATGGACCGCATTAAGCGAGTCACAGAAGGACAGTTCCtt                                                                                                                                                                                       >  1:334379/1‑301 (MQ=255)`  
                                                                                                                `ccTATCGCTATATCTGGCGCTCAGGCTATCAGTGATCAGAACGCTCAGGCCAAAATGATTGCCGCTAAGACCGCTGCTGGTCGTCGTCAAGCTATGGAAATCATGAGGCAGACGAACATCCAGAATGCTGACCTATCGTTGCAAGCTCGAAGTAAACTTGAGGAAGCGTCCGCCGAGTTGACCCCACAGAACATGCAGAAGGTCCAAGCTATTGGGTCTATCCGAGCGGCTATCGGATAGAGTATGCTTGAAGGTTCCTCAATGGACAGCATTAAGCGAGTCACAGAAGGACAGTTCATTc                                                                                                                                                                                      >  1:56143/1‑301 (MQ=255)`  
                                                                                                                   `aTCGCTATATCTGGCGCTCAGGCTATCAGTGATCAGAACGCTCAGGCCAAAATGATTGCCGCTCAGACCGCTGCTGGTCGTCGTCAAGCTATGGAAATCATGAGGCAGACGAACATCCAGAATGCTCACCTATCGTTGCAAGCTCGAAGTAAAATTGAGGAAGCGTCCGCCGAGTTGACCCCACAGAACATGCAGCAGGTCCAAGCTATTGGTTCTATCCGAGCGGCTATCGGAGAGAGTATGCTTGAAGTTTCCTCAATGGACCGCATTAAGCTAGTCACAGCATGACAGTTCATTCggg                                                                                                                                                                                   >  1:19828/1‑301 (MQ=255)`  
                                                                                                                         `atatCTGGCGCTCAGGCTATCAGTGATCAGAACGCTCAGGCCAAAATGATTGCCGCTCAGACCGCTGCTCGTCGTCGTCAAGCTATGGAAATCATGAGTCAGACGAACATCCAGAATGCTGACCTATCGTTGCAAGCTCGAAGTAAACTTGAGGAAGCGTCCGCCGAGTTGACCCCACAGAACATGCAGAAGGTCCAAGCTATTGGGTCTATCCGAGCGGCTATCGGAGAGAGTATGCTTGAAGGTTCCTCAATGGACCGCATTAAGCGAGTTACAGactgtctcttctacacatctccga                                                                                                                                                                             >  1:293670/1‑278 (MQ=255)`  
                                                                                                                           `atCTGGCGCTCAGGCTATCAGTGGTCAGAACGCTCAGGCCAAAATGATTGCCGCTCAGACCGCTGCTCGTCGTCGTCAAGCTATGGAAATCATGAGGCAGACGAACATCCAGAATGCTGACCTATCGTTGCAAGCTCGAAGTAAACTTGAGGAAGCGTCCGCCGAGTTGACCCCACAGAACATGCAGAAGGTCCAAGCTATTGGGTCTATCCGAGCGGCTATCGGAGAGAGTATGCTTGAAGGTTCCTCAATGGACCGCATTAAGCGAGTCACAGAAGGAcctgtctcttatacacatctc                                                                                                                                                                           >  1:207823/1‑281 (MQ=255)`  
                                                                                                                           `atCTGGCGCTCAGGCTATCAGTGATCAGAACGCTCAGGCCAAAATGATTGCCGCTCAGACCGCTGCTCGTCGTCGTCAAGCTATGGAAATCATGAGGCAGACGAACATCCAGAATGCTGACCTATCGTTGCAAGCTCGAAGTAAACTTGAGGAAGCGTCCGCCGAGTTGACCCCACAGAACATGCAGAAGGTCCAAGCTATTGGGTCTATCCGAGCGGCTATCGGAGAGAGTATGCTTGAAGGTTCCTCAATGGACCGCATTAAGCGAGTCACAGAAGGACAGTTCATTCGGGactgtctc                                                                                                                                                                           >  1:165314/1‑294 (MQ=255)`  
                                                                                                                           `atCTGGCGCTCAGGCTATCAGTGATCAGAACGCTCAGGCCAAAATGATTGCCGCTCAGACCGCTGCTCGTCGTCGTCAAGCTATGGAAATCATGAGGCAGACGAACATCCAGAATGCTGACCTATCGTTGCAAGCTCGAAGTAAACTTGAGGAAGCGTCCGCCGAGTTGACCCCACAGAACATGCAGAAGGTCCAAGCTATTGGGTCTATCCGAGCGGCTATCGGAGAGAGTATGCTTGAAGGTTCCTCAATGGACCGCATTAAGCGAGTCACAGAAGGACAGTTCATTCGGGAAGCCAat                                                                                                                                                                           >  1:131543/1‑301 (MQ=255)`  
                                                                                                                           `atCTGGCGCTCAGGCTATCAGTGATCAGAACGCTCAGGCCAAAATGATTGCCGCTCAGACCGCTGCTCGTCGTCGTCAAGCTATGGAAATCATGAGGCAGACGAACATCCAGAATGCTGACCTATCGTTGCAAGCTCGAAGTAAACTTGAGGAAGCGTCCGCCGAGTTGACCCCACAGAACATGCAGAAGGTCCAAGCTATTGGGTCTATCCGAGCGGCTATCGGAGAGAGTATGCTTGAAGGTTCCTCAATGGACCGCATTAAGCGAGTCACAGAAGGACAGTTCATTCGGGAAGCCAat                                                                                                                                                                           >  1:267367/1‑301 (MQ=255)`  
                                                                                                                              `tGGCGCTCAGGCTATCAGTGATCAGAACGCTCAGGCCAAAATGATTGCCGCTCAGACCGCTGCTCGTCGTCGTCAAGCTATGGAAATCATGAGGCAGACGAACATCCAGAATGCTGACCTATCGTTGCAAGCTCGAAGTAAACTTGAGGAAGCGTCCGCCGAGTTGACCCCACAGAACATGCAGAAGGTCCAAGCTATTGGGTCTATCCGAGCGGCTATCGGAGAGAGTATGCTTGAAGGTTCCTCAATGGACCGCATTAAGCGAGTCACAGAAGGACAGTTCATTCGGGAAGCCAATATg                                                                                                                                                                        <  1:331843/301‑1 (MQ=255)`  
                                                                                                                              `cggaagtgtgtataagagacagcAGAACGCTCAGTCCAAAATGAGTGCCGCTCAGACCGCTGCTGGTCGTCGTCAAGCTATGGAAATCATGAGGCAGACGAACATCCAGAATGCTGACCTATCGTTGCAAGCTCGAAGTAAACTTGAGGAAGCGTCCGCCGAGTTGACCCCACAGAACATGCAGAAGGTCCAAGCTATTGGGTCTATCCGAGCGGCTATCGGAGAGAGTATGCTTGAAGGTTCCTCAATGGACCGCATTAAGCGAGTCACAGAAGGACAGTTCATTCGGGAAGCCAATATg                                                                                                                                                                        <  1:253954/279‑1 (MQ=255)`  
                                                                                                                               `ggCGCTCAGGCTATCAGTGATCAGAACGCTCAGGCCAAAATGATTGCCGCTCAGACCGCTGCTCGTCGTCGTCAAGCTATGGAAATCATGAGGCAGACGAACATCCAGAATGCTGACCTATCGTTGCAAGCTCGAAGTAAACTTGAGGAAGCGTCCGCCGAGTTGACCCCACAGAACATGCAGAAGGTCCAAGCTATTGGGTCTATCCGAGCGGCTATCGGAGAGAGTATGCTTGAAGGTTCCTCAATGGACCGCATTAAGCGAGTCACAGAAGGACAGTTCATTCTGGAAGCCAATATgg                                                                                                                                                                       <  1:1221/301‑1 (MQ=255)`  
                                                                                                                               `ggCGCTCAGGCTATCAGTGATCAGAACGCTCAGGCCAAAATGATTGCCGCTCAGACCGCTGATGGTCGTCGTCAAGCTATGGAAATCATGAGGCAGACGAACATCCAGAATGCTGACCTATCGTTGCAAGCTCGAAGTAAACTTGAGGAAGCGTCCGCCGAGTTGACCCCACAGAACATGCAGAAGGTCCAAGCTATTGGGTCTATCCGAGCGGCTATCGGAGAGAGTATGCTTGAAGGTTCCTCAATGGACCGCATTAAGCGAGTCACAGAAGGACAGTTCATTCGGGAAGCCAATATgg                                                                                                                                                                       <  1:225849/301‑1 (MQ=255)`  
                                                                                                                                  `gcTCAGGCTATCCGTGATGAGAACGCTCAGGCCAAAATGATTGCCGCTCAGACCGCTGCTGGTCGTCGTCAAGCTATGGAAATCATGAGGCAGACGAACATCCAGAATGCTGACCTATCGTTGCAAGCTCGAAGTAAACTTGAGGAAGCGTCCGCCGAGTTGACCCCACAGAACATGCAGAAGGTCCAAGCTATTGGGTCTATCCGAGCGGCTATCGGAGAGAGTATGCTTGAAGGTTCCTCAATGGACCGCATTAAGCGAGTCACAGAAGGACAGTTCATTCGGGAAGCCAATATGGTaa                                                                                                                                                                    <  1:9626/301‑1 (MQ=255)`  
                                                                                                                                     `cAGGCTATCAGTGATCAGAACGCTCAGGCCAAAATGATTGCCGCTCAGACCGCTGCTCGTCGTCGTCAAGCTATGGAAATCATGAGGCAGACGAACATCCAGAATGCTGACCTATCGTTGCAAGCTCGAAGTAAACTTGAGGAAGCGTCCGCCGAGTTGACCCCACAGAACATGCAGAATGTCCAAGCTATTGGGTCTATCCGAGCGGCTATCGGAGAGAGTATGCTTGAAGGTTCCTCAATGGACCGCATTAAGCGAGTCACAGAAGGACAGTTCATTCGGGAAGCCAATATGGTAACTg                                                                                                                                                                 <  1:6986/301‑1 (MQ=255)`  
                                                                                                                                       `ggCTATCAGTGATCAGAACGCTCAGGCCAAAATGATTGCCGCTCAGACCGCTGCTGGTCGTCGTCAAGCTATGGAAATCATGAGGCAGACGAACATCCAGAATGCTGACCTATCGTTGCAAGCTCGAAGTAAACTTGAGGAAGCGTCCGCCGAGTTGACCCCACAGAACATGCAGAAGGTCCAAGCTATTGGGTCTATCCGAGCGGCTATCGGAGAGAGTATGCTTGAAGGTTCCTCAATGGACCGCATTAAGCGAGTCACAGAAGGACAGTTCATTCGGGAAGCCAATATGGTAACTgag                                                                                                                                                               >  1:228999/1‑301 (MQ=255)`  
                                                                                                                                         `cTATCAGTGATCAGAACGCTCAGGCCAAAATGATTGCCGCTCAGACCGCTGCTGGTCGTCGTCAAGCTATGGAAATCATGAGGCAGACGAACATCCAGAATGCTGACCTCTCGTTGCAAGCTCGAAGTAAACTTGAGGAAGCGTCCGCCGAGTAGACCCCACAGAACATGCAGAAGGTCCAAGCTATTGGGTCTATCCGAGCGGCTATCGGAACGAGTATGCTTGAAGGTTCCTAAATGGACCGCATTAAGCGAGTCACAGAAGGACAGTTCATTCGGGAAGCCAATATGGTAACTGAGaa                                                                                                                                                             >  1:261332/1‑301 (MQ=255)`  
                                                                                                                                            `tCAGTGATCAGAACGCTCAGGCCAAAATGATTGCCGCTCAGACCGCTGCTCGTCGTCGTCAAGCTATGGAAATCATGAGGCAGACGAACATCCAGAATGCTGACCTATCGTTGCAAGCTCGAAGTAAACTTGAGGAAGCGTCCGCCGAGTTGACCCCACAGAACATGCAGAAGGTCCAAGCTATTGGGTCTATCCGAGCGGCTATCGGAGAGAGTATGCTTGAAGGTTCCTCAATGGACCGCATTAAGCGAGTCACAGAAGGACAGTTCATTCGGGAAGCCAATATTGTAACTGAGAACTa                                                                                                                                                          >  1:3164/1‑301 (MQ=255)`  
                                                                                                                                             `cAGTGATCAGAACGCTCAGGCCAAAATGATTGCCGCTCAGACCGCTGCTGGTCGTCGTCAAGCTATGGAAATCATGAGGCAGACGAACATCCAGAATGCTGACCTATCGTTGCAAGCTCGAAGTAAACTTGAGGAAGCGTCCGCCGAGTTGACCCCACAGAACATGCAGAAGGTCCAAGCTATTGTGTCTATCCGAGCGGCTATCGGAGAGAGTATGCTTGAAGGTTCCTCAATGGACCGCATTAAGCGAGTCACAGAAGGACAGTTCATTCGGGAAGCCAATATGGTAACTGAGAACTAt                                                                                                                                                         <  1:178813/301‑1 (MQ=255)`  
                                                                                                                                              `aGTGATCAGAACGCTCAGGCCAAAATGATTGCCGCTCAGACCGCTGCTCGTCGTCGTCAAGCTATGGAAATCATGAGGCAGACGAACATCCAGAATGCTGACCTATCGTTGCAAGCTCGAAGTAAACTTGAGGAAGCGTCCGCCGAGTTGAACCCACAGAACATGCAGAAGGTCCAAGCTATTGGGTCTATCCGAGCGGCTATCGGAGAGAGTATGCTTGAAGGTTCCTCAATGGACCGCATTAAGCGAGTCACAGAAGGACAGTTCATTCGGGAAGCCAatatctgtctcttatacacat                                                                                                                                                        >  1:275515/1‑284 (MQ=255)`  
                                                                                                                                               `gTGATCAGAACGCTCAGGCCAAAATGATTGCCGCTCAGACCGCTGCTGGTCGTCGTCAAGCTATGGAAATCATGAGGCAGACGAACATCCAGAATGCTGACCTATCGTTGCAAGCTCGAAGTAAACTTGAGGAAGCGTCCGCCAAGTTGACCCCACAGAACATGCAGAAGGTCCAAGCTATTGGGTCTATCCGAGCGGCTATCGGAGAGAGTATGCTTGAAGGTTCCTCAATGGACCGCATTAAGCGAGTCACAGAAGGACAGTTCATTCGGGAAGCCAATATGGTAACTGAGACCTATct                                                                                                                                                       >  1:52697/1‑300 (MQ=255)`  
                                                                                                                                                 `gatCAGAACGCTCAGGCCAAAATGATTGCCGCTCAGACCGCTGCTCGTCGTCGTCAAGCTATGGAAATCATGAGGCAGACGAACATCCAGAATGCTGACCTATCGTTGCAAGCTCGAAGTAAACTTGAGGAAGCGTCCGCCGAGTTGACCCCACAGAACATGCAGAAGGTCCAAGCTATTGGGTCTATCCGAGCGGCTATCGGAGAGAGTATGCTTGAAGGTTCCTCAATGGACCGCATTAAGCGAGTCACAGAAGGACAGTTCATTCGGGAAGCCAATATGGTAACTGAGAACTATCGcc                                                                                                                                                     >  1:324441/3‑301 (MQ=255)`  
                                                                                                                                                    `cAGAATGCTCAGGCCAAAATGATTGCCGCTCAGACCGCTGCTGGTCGTCGTCAAGCTATGGAAATCATGAGGCAGACGAACATCCAGAATGCTGACCTATCGTTGCAAGCTCGAAGTAAACTTGAGGAAGCGTCCGCCGAGTTGACCCCACAGAACATGCAGAAGGTCCAAGCTATTGGGTCTATCCGAGCGGCTATCGGAGAGAGTATGCTTGAAGGTTCCTCAATGGACCGCATTAAGCGAGTCACAGAAGGACAGTTCATTCGGGAAGCCAATATGGTCACTGAGAACTATCGCCGTg                                                                                                                                                  >  1:204408/1‑301 (MQ=255)`  
                                                                                                                                                    `cAGAACGCTCAGGCCAAAATGATTGCCGCTCAGACCGCTGCTGGTCGTCGTCAAGCTATGGAAATCATGAGGCAGACGAACATCCAGAATGCTTACCTATCGTTGCAAGCTCGAAGTAAACTTGAGGAAGCGTCCGCCGAGTTGACCCCACAGAACATGCAGAAGGTCCAAGCTATTGGGTCTATCCGAGCGGCTATCGGAGAGAGTATGCTTGAAGGTTCCTCAATGGACCGCATTAAGCGAGTAACAGAAGGACAGTTCATTCGGGAAGCCAATATGGTAACTGAGAACTATCGCCGTg                                                                                                                                                  >  1:211148/1‑301 (MQ=255)`  
                                                                                                                                                              `aGGCCAAAATGATTGCCGCTCAGACCGCTGCTCGTCGTCGTCAAGCTATGGAAATCATGAGGCAGACGAACATCCAGAATGCTGACCTATCGTTGCAAGCTCGAAGTAAACTTGAGGAAGCGTCCGCCGAGTTGACCCCACAGAACATGCAGAAGGTCCAAGCTATTGGTTCTATCCGAGCGGCTATCGGAGAGAGTATGCTTGAAGGTTCCTCAATGGACCGCATTAAGCGAGTCACAGAAGGACAGTTCATTCGGGAAGCCAATATGGTAACTGAGAACTATCGCCGTGACTACCAAGc                                                                                                                                        <  1:132823/301‑1 (MQ=255)`  
                                                                                                                                                                `gCCAAAATGATTGCCGCTCAGACCGCTGCTCGTCGTCGTCAAGCTATGGAAATCATGAGGCAGACGAACATCCAGAATGCTGACCTATCGTTGCAAGCTCGAAGTAAACTTGAGGAAGCGTCCGCCGAGTTGACCCCACAGAACATGCAGAAGGTCCAAGCTATTGGGTCTATCCGAGCGGCTATCGGAGAGAGTATGCTTGAAGGTTCCTCAATGGACCGCATTAAGCGAGTCACAGAAGTACAGTTCATTCGGTAAGCCAATATGGTAACTGAGAACTATCGCCGTGACTACCAAGCaa                                                                                                                                      <  1:232309/301‑1 (MQ=255)`  
                                                                                                                                                                         `aTTGCCGCTCAGACCGCTGCTCGTCGTCGTCAAGCTATGGAAATCATGAGGCAGACGAACATCCAGAATGCTGACCTATCGTTGCAAGCTCGAAGTAAACTTGAGGAAGCGTCCGCCGAGTTGACCCCACAGAACATGCAGAAGGTCCAAGCTATTGGGTCTATCCTAGCGGCTATCGGAGAGAGTATGCTTGAAGGTTCCTCAATGGACCGCATTAAGCGAGTCACAGAAGGACAGTTCATTCGGGAAGCCAATATGGTAACTGAGAACTATCGCCGTGACTACCAAGCAATCTTCGcac                                                                                                                             <  1:289534/301‑1 (MQ=255)`  
                                                                                                                                                                           `tGCCGCTCAGACCGCTGCTGGTCGTCGTCAAGCTATGGAAATCATGAGGCAGACGAACATCCAGAATGCTGACCTATCGTTGCAAGCTCGAAGTAAACTTGAGGAAGCGTCCGCCGAGTTGACCCCACAGAACATGCAGAAGGTCCAAGCTATTGGGTCTATCCGAGCGGCTATCGGAGAGAGTATGCTTGAAGGTTCCTCAATGGACCGCATTAAGCGAGTCACAGAAGGACAGTTCATTCGGGAAGCCAATATGGTAACTGAGAACTATCGCCGTGACTACCAAGCAATCTTCGCACAg                                                                                                                           <  1:156974/301‑1 (MQ=255)`  
                                                                                                                                                                             `ccGCTCATACCGCTGCTGGTCGTCGTCAAGCTATGGAAATCATGAGGCAGACGAACATCCAGAATGCTGACCTATCGTTGCAAGCTCGAAGTAAACTTGAGGAAGCGTCCGCCGAGTTGACCCCACAGAACATGCAGCAGGTCCAAGCTATTGGGTCTATCCGAGCGGCTATCGGAGAGAGTATGCTTGAAGGTTCCTCAATGGACCGCATTAAGCGAGTCACAGAAGGACAGTTCATTCGGGAAGCCAATTGGGTAACTGAGAACTATCGCCGTTACTACCAAGCAATCTTCGCCAAGCa                                                                                                                         >  1:145571/1‑301 (MQ=255)`  
                                                                                                                                                                               `gcgcAGACCGCTGCTGGTCGTCGTCAAGCTATGGAAATCATGAGGCAGACGAACATCCAGAATGCTGACCTATCGTTGCAAGCTCGAAGTAAACTTGAGGAAGCGTCCGCCGAGTTGACCCCACAGAACATGCAGAAGGTCCAAGCTATTGGGTCTATCCGAGCGGCTATCGGAGAGAGTATGCTTGAAGGTTCCTCAATGGACCGCATGAAGCGAGTCACAGAAGGACAGTTCATTCGGGAAGCCAATATGGTAACTGAGAACTATCGCCGTGACTACCAAGCAATCTTCGCACAGCAAc                                                                                                                       <  1:202028/298‑1 (MQ=255)`  
                                                                                                                                                                                    `gACCGCTGCTGGTCGTCGTCAAGCTATGGAAATCATGAGGCAGACGAACATCCAGAATGCTGACCTATCGTTACAAGCTCGAAGTAAACTTGAGGAAGCGTCCGCCGAGTTGACCCCACAGAACATGCAGAAGGTCCAAGCTATTGGGTCTATCCGAGCGGCTATCGGAGAGAGTATGCTTGAAGGTTCCTCAATGGACCGCATTAAGCGAGTCACAGAAGTACAGTTCATTCGGGAAGCCAATATGGTAACTGAGAACTATCGCCGTGACTACCAAGCAATCTTCGCACAGCACCTtggt                                                                                                                  >  1:89900/1‑301 (MQ=255)`  
                                                                                                                                                                                    `gACCGCTGCTCGTCGTCGTCAAGCTATGGAAATCATGAGGCAGACGAACATCCAGAATGCTGACCTATCGTTGCAAGCTCGAAGTAAACTTGAGGAAGCGTCCGCCGAGTTGACCCCACAGAACATGCAGAAGGTCCAAGCTATTGGGTCTATCCGAGCGGCTATCGGAGAGAGTATGCTTGAAGGTTACTCAATGGACCGCATTAAGCGAGTAACAGAAGGACAGTTCATTCGGGAAGCCAATATGGTAACTGAGAACTATCGCCGTGACTACCAAGCAATCTTCGCACAGCAACTtggt                                                                                                                  >  1:127919/1‑301 (MQ=255)`  
                                                                                                                                                                                         `ctgcttGTCGTCGTCAAGCTATGGAAATCATGAGGCAGACGAACATCCAGAATGCTGACCTATCGTTGCAAGCTCGAAGTAAACTTGAGGAAGCGTCCGCCGAGTTGACCCCACAGAACATGCAGAAGGTCCAAGCTATTGGGTCTATCCGAGCGGCTATCGGAGAGAGTATGCTTGAAGGTTCCTCAATGGACCGCATTAAGCGAGTCACAGAAGGACAGTTCATTCGGGAAGCCAATATGGTAACTGAGAACTATCGCCGTGACTACCAAGCAATCTTCGCACAGCAACTtggtgggat                                                                                                             >  1:195656/1‑298 (MQ=255)`  
                                                                                                                                                                                           `gctcGTCGTCGTCAAGCTATGGAAATCATGAGGCAGACGAACATCCAGAATGCTGACCTATCGTTGCAAGCTCGAAGTAAACTTGAGGAAGCGTCCGCCGAGTTGACCCCACAGAACATGCAGAAGGTCCAAGCTATTGGGTCTATCCGAGCGGCTATCGGAGAGAGTATGCTTGAAGGTTCCTCAATGGACCGCATTAAGCGAGTCACAGAAGGACAGTTCATTCGGGAAGCCAATATGGTAACTGAGAACTATCGCCGTGACTACCAAGCAATCTTCGCACAGCAACTTGGTGGTACTc                                                                                                           <  1:304120/301‑1 (MQ=255)`  
                                                                                                                                                                                           `aatgtgtataagagacagaTGGAAAGCATGAGGCAGACGAACATACAGAATGCTGACCTATCGTTGCAAGCTCGAAGTAAACTTGAGGAAGCGTCCGCCGAGTTGACCCCACAGAACATGCAGAAGGTCCAAGCTATTGGGTCTATCCGAGCGGCTATCGGAGAGAGTATGCTTGAAGGTTCCTCAATGGACCGCATTAAGCGAGTCACAGAAGGACAGTTCATTCGGGAAGCCAATATGGTAACTGAGAACTATCGCCGTGACTACCAAGCAATCTTCGCACAGCAACTTGGTGGTACTc                                                                                                           <  1:24684/283‑1 (MQ=255)`  
                                                                                                                                                                                                  `gtcgtcAAGCTATGGAAATCATGAGGCAGACGAACATCCAGAATGCTGACCTATCGTTGCAAGCTCGAAGTAAACTTGAGGAAGCGTCCGCCGAGTTGACCCCACAGAACATGCAGAAGGTCCAAGCTATTGGGTCTATCTGAGCGGCTATCGGAGAGAGTATGCTTGAAGGTTCCTCAATGGACCGCATTAAGCGAGTCACAGAAGGACAGTTCATTCGGGAAGCCAATATGGTAACTGAGAACTATCGCCGTGACTACCAAGCAATCTTCGCACAGCAACTTGGTGGTACTCAAAGtgc                                                                                                    <  1:124666/301‑1 (MQ=255)`  
                                                                                                                                                                                                   `tcgtcAAGCTATGGAAATCATGAGGCAGACGAACATCCAGAATGCTGACCTATCGTTGCAAGCTCGAAGTAAACTTGAGGAAGCGTCCGCCGAGTTGACCCCACAGAACATGCAGAAGGTCCAAGCTATTGGGTCTATCCGAGCGGCTATCGGAGAGAGTATGCTTGAAGGTTCCTCAATGGACCGCATTAAGCGAGTCACAGAAGGACAGTTCATTCGGGAAGCCAATAAGGTAACTGAGAACTATCGCCGTGACTACCAAGCAATCTTCGCACAGCAACTTGGTGGTACTCAACGtgct                                                                                                   >  1:141176/1‑301 (MQ=255)`  
                                                                                                                                                                                                   `ggacagaGCTATGGAAATCATGAGGCATACGAACATCCAGAATGCTGACCTATCGTTGCAAGCTCGAAGTAAACTTGAGGAAGCGTCCGCCGAGTTGACCCCACAGAACATGCAGAAGGTCCAAGCTATTGGGTCTATCCGAGCGGCTATCGGAGAGAGTATGCTTGAAGGTTCCTCAATGGACCGCATTAAGCGAGTCACAGAAGGACAGTTCATTCGGGAAGCCAATATGGTAACTGAGAACTATCGCCGTGACTACCAAGCAATCTTCGCACAGCAACTTGGTGGTACTCAAAGtgct                                                                                                   <  1:264456/295‑1 (MQ=255)`  
                                                                                                                                                                                                          `gCTATGGACATCATGATGCAGACGACCATCCAGAATGCTGACCTATCGTTGCAAGCTCGAATTAAACTTGAGGAAGCGTCCGCCGAGTTGACCCCACAGAACATGCAGAAGTTCCAAGCTATTGGGTCTATCCGAGCGGCTATCGGAGAGAGTATGCTTGAAGGTTCCTCAATGGACCGCATTAAGCGAGTCACAGAAGGACAGTTCATTCGGGAAGCCAATATGGTAACTGAGAACTATCGCCGTGACTACCAAGCAATCTTCGCACAGCAACTTTGTGGTACTCAAAGTGCTGCAAGTc                                                                                            <  1:161933/301‑1 (MQ=255)`  
                                                                                                                                                                                                                `gAAATCATGAGGCAGACGAACATCCAGAATGCTGACCTATCGTTGCAAGCTCGAAGTAAACTTGAGGAAGCGTCCGCCGAGTTGACCCCACAGAACATGCAGAAGGTCCAAGCTATTGGGTCTATCCGAGCGGCTATCGGAGAGAGTATGCTTGAAGGTTCCTCAATGGACCGCATTAAGCGAGTCACAGAAGGACAGTTCATTCGGGAAGCCAATATGGTAACTGAGAACTATCGCCGTGACTACCAAGCAATCTTCGCACAGCAACTTGGTGGTACTCAAAGTGCTGCaactgtctctt                                                                                      >  1:336978/1‑292 (MQ=255)`  
                                                                                                                                                                                                                          `ggCAGACGAACATCCAGAATGCTGACCTATCGTTGCAAGCTAGAAGTAAACTTGAGGAAGCGTCCGCCGAGTTGACCCCACAGAACATGCAGAAGGTCCAAGCTATTGGGTCTATCCGAGCGGCTATCGGAGAGAGTACGCTTGAAGGTTCCTCAATGGACCGTATTAAGCGAGTCACAGAAGGACAGTTCATTCGGGAAGCCAATATGGTAACTGAGAACTATCGCCGTGACTACCAAGCAATCTTCTCACAGCAACTTGGTGGTACTCAAAGTGCTTCaactgtctcttatacacatct                                                                            >  1:28217/1‑282 (MQ=255)`  
                                                                                                                                                                                                                              `gACGAACATCTAGAATGCTGACCTATCGTTGCAAGCTCGAAGTAAACTTGAGGAAGCGTCCGCCGAGTTGACCCCACAGAACATGCAGAAGGTCCAAGCTATTGGGTCTATCCGAGCGGCTATCGGAGAGAGTATGCTTGAAGGTTCCTCAATGGACCGCATTAAGCGAGTCACAGAAGGACAGTTCATTCGGGAAGCCAATATGGTAACTGAGAACTATCGCCGTGACTACCAAGCAATCTTCGCACAGCAACTTGGTGGTACTCAAAGTGCTGCAAGTCAGATTGACGAAATCTATAag                                                                        <  1:107308/301‑1 (MQ=255)`  
                                                                                                                                                                                                                                    `cATCCAGAATGCTGACCTATCGTTGCAAGCTCGAAGTAAACTTGAGGAAGCGTCCGCCGAGTTGACCCCACAGAACATGCAGAAGGTCCAAGCTATTGGGTCTATCCGAGCGGCTATCGGAGAGAGTATGCTTGAAGGTTCCTCAATGGACCGCATTAAGCGAGTCACAGAAGGACAGTTCATTCGGGAAGCCAATATGGTAACTGAGAACTATCGCCGTGACTACCAAGCAATCTTCGCACAGCAACTTGGTGGTACTCAAAGTGCTGCAAGTCAGATTGACGAAATCTATAAGAGCGaa                                                                  >  1:40875/1‑301 (MQ=255)`  
                                                                                                                                                                                                                                      `tataagagacagACCTATCGTTGCAAGCTCGAAGTAAACTTGAGGAAGCGTCCGCCGAGTTGACCCCACAGAACATGCAGAAGGTCCAAGCTATTGGGTCTATCCGATCGGCTATCGGAGAGAGTATGCTTGAAGGTTCCTCAATGGACCGCATTAAGAGAGTCACAGAAGGACAGTTCATTCGGGAAGCTAATATGGTAACTGAGAACTATCGACGTGACTACCAAGCAATCTTCGCACAGCAACTTGGTGGTACTCAAAGTGCTGCAAGTCAGATTGACGAAATCTATAAGAGCGaaca                                                                <  1:319469/290‑1 (MQ=255)`  
                                                                                                                                                                                                                                          `gAATGCTGACCTATCGTTGCAAGCTCGAAGTAAACTTGAGGAAGCGTCCGCCGAGTTGACCCCACAGAACATGCAGAAGGTCCAAGCTATTGGGTCTATCCGAGCGGCTATCGGAGAGAGTATGCCTGAAGGTTCCTCAATGGACCGCATTAAGCGAGTCACAGAAGGACAGTTCATTCGGGAAGCCAATATGGTAACTGAGAACTATCGCCGTGACTACCAAGCAATCTTCGCACAGCAACTTGGTGGTACTCAAAGTGCTGCAAGTCAGATTGACGAAATCTATAAGAGctgtctctta                                                            >  1:217175/1‑292 (MQ=255)`  
                                                                                                                                                                                                                                           `agacaggACCTATCGTTGCAAGCTCGAAGTAAACTTGAGGAAGCGTCCGCCGAGTTGACCCCACAGAACATGCAGAAGGTCCAAGCTATTGGGTCTATCCGAGCGGCTATCGGAGAGAGTATGCTTGAAGGTTCCTCAATGGACCGCATTAAGCGAGTCACAGAAGGACAGTTCATTCGGGAAGCCAATATGGTAACTGAGAACTATCGCCGTGACTACCAAGCAATCTTCGCACAGCAACTTGGTGGTACTCAAAGTGCTGCAAGTCAGATTGACGAAATCTATAAGAGCGaacagaaac                                                           <  1:116754/295‑1 (MQ=255)`  
                                                                                                                                                                                                                                                    `cTATCGTTGCAAGCTCGAAGTAAACTTGAGGAAGCGTCCGCCGAGTTGACCCCACAGAACATGCAGAAGGTCCAAGCTATTGGGTCTATCCGAGCGGCTATCGGCGAGAGTATGCTTGAAGGTTCCTCAATGGACCGCATTAAGCGAGTCACAGAAGGACAGTTCATTCGTGAAGCCAATCTGGTAACTGAGAACTATCGCCGTGACTACCAAGCAATCTTCGCACAGCAACTTGGTGGTACTCAAAGTGCTGCAAGTCATATTCACGAAATCTATAAGAGCGAACAGAAACAGAAGAGTa                                                  >  1:213193/1‑301 (MQ=255)`  
                                                                                                                                                                                                                                                      `aTCGTTGCAAGCTCGAAGTAAACTTGAGGAAGCGTCCGCCGAGTTGACCCCACAGAACATGCAGAAGGTCCAAGCTATTGGGTCTATCCGAGCGGCTATCGGAGAGAGTATGCTTGAAGGTTCCTCAATGGACCGCATTAAGCGAGTCACAGAAGGACAGTTCATTCGGGAAGCCAATATGGTAACTGAGAACTATCGCCGTGACTACCAAGCAATCTTCGCACAGCAACTTGGTGGTACTCAAAGTGCTCCAAGTCAGATTGACGAAATCTATAAGAGCGAACAGAAACAGAAGAGTAAg                                                >  1:300853/1‑301 (MQ=255)`  
                                                                                                                                                                                                                                                              `aaGCTCGAAGTAAACTTGAGGAAGCGTCCGCCGAGTTGACCCCACAGAACATGCAGAAGGTCCAAGCTATTGGGTCTATCCGAGCGGCTATCGGAGAGAGTATGCTTGAAGGTTCCTCAATGGACCGCATTAAGCGAGTCACAGAATGACAGTTCATTCGGGAAGCCAATATGGTAACTGAGAACTATCGCCGTGACTACCAAGCAATCTTCGCACAGCAACTTGGTGGTACTCAAAGTGCTGCAAGTCAGATTGACGAAATCTATTAGAGCGAACAGAAACAGAAGAGTAAGCTACAGAt                                        <  1:82575/301‑1 (MQ=255)`  
                                                                                                                                                                                                                                                              `aaGCTCGAAGTAAACTTGAGGAAGCGTCCGCCGAGTTGACCCCACAGAACATGCAGAAGGTCCAAGCTATTGGGTCTATCCGAGCGGCTATCGGAGAGAGTATGCTTGAAGGTTCCTCAATGGACCGCATTAAGCGAGTCACAGAAGGACAGTTCATTCGGGAAGCCAATATGGTAACTGAGAACTATCGCCGTGACTACCAAGCAATCTTCGCACAGCAACTTGGTGGTACTCAAAGTGCTGCAAGTCAGATTGACGAAATCTATAAGAGCGAACAGAAACAGAAGAGTAAGCTACAGAt                                        <  1:237871/301‑1 (MQ=255)`  
                                                                                                                                                                                                                                                                     `aaGTAAACTTGAGGAAGCGTCCGCCGAGTTGACCCCACAGAACATGCAGAAGGTCCAAGCTATTGGGTCTATCCGAGCGGCTATCGGAGAGAGTATGCTTGAAGGTTCCTCAATGGACCGCATTAAGCGAGTCACAGAAGGACAGTTCATTCGGGAAGCCAATATGGTAACTGAGAACTATCGCCGTGACTACCAAGCAATCTTCGCACAGCAACTTGGTGGTACTCAAAGTGCTGCAAGTCAGATTGACGAAATCTATAAGAGCGAACAGAAACAGAAGAGTAAGCTACAGATGGTTCTg                                 >  1:256189/1‑301 (MQ=255)`  
                                                                                                                                                                                                                                                                     `aaGTAAACTTGAGGAAGCGTCCGCCGAGTTGACCCCACAGAACATGCAGAAGGTCCAAGCTATTGGGTCTATACGAGCGGCTATCGGAGAGAGTATGCTTGAAGGTTCCTCAATGGACCGCATTAAGCGAGTCACAGAAGGACAGTTCATTCGGGAAGCCAATATGGTAACTGAGAACTATCGCCGTGACTACCAAGCAATCTTCGCAAAGCAACTTGGTGGTACTCAAAGTTCTGCAAGTCAGATTGACGAAATCTATAAGAGCGAACAGAAACAGAAGAGTAAGATACAGATGGCTCTg                                 >  1:154174/1‑301 (MQ=255)`  
                                                                                                                                                                                                                                                                              `tGAGGAAGCGTCCGCCGAGTTGACCCCACAGAACATGCAGAAGGTCCAAGCTATTGGGTCTATCCGAGCGGCTATCGGAGAGAGTATGCTTGAAGGTTCCTCAATGGACCGCATTAAGCGAGTCACAGAAGGACAGTTCATTCGGGAAGCCAATATGGTAACTGAGAACTATCGCCGTGACTACCAAGCAATCTTCGCACAGCAACTTGGTGGTACTCAAAGTGCTGCAAGTCAGATTGACGAAATCTATAAGAGCGAACAGAAACAGAAGAGTAAGCTACAGATGGTTCTGGACCCACTg                        <  1:307877/301‑1 (MQ=255)`  
                                                                                                                                                                                                                                                                                 `ggAAGCGTCCGCCGAGTTGACCCCACAGAACATGCAGAAGGTCCAAGCTATTGGGTCTATCCGAGCGGCTATCGGAGAGAGTATGCTTGAAGGTTCCTCAATGGACCGCATTAAGCGAGTCACAGAAGGACAGTTCATTCGGGAAGCCAATATGGTAACTGAGAACTATCGCCGTGACTACCAAGCAATCTTCGCACAGCAACTTGGTGGTACTCAAAGTGCTGCAAGTCAGATTGACGAAATCTATAAGAGCGAACAGAAACAGAAGAGTAAGCTACAGATGGTTCTGGACCCACTGGCt                     <  1:342174/301‑1 (MQ=255)`  
                                                                                                                                                                                                                                                                                    `aGCGTCCGCCGAGTTGACCCCACAGAACATGCAGAAGGTCCAAGCTATTGGGTCTATCCGAGCGGCTATCGGAGAGAGTATGCTTGAAGGTTCCTCAATGGACCGCATTAAGCGAGTCACAGAAGGACAGTTCATTCGGGAAGCCAATATGGTAACTGAGAACTATCGCCGTGACTACCAAGCAATCTTCGCACAGCAACTTGGTGGTACTCAAAGTGCTGCAAGTCAGATTGACGAAATCTATAAGAGCGAACAGAAACAGAAGAGTAAGCTACAGATGGTTCTGGACCCACTGGCTATc                  >  1:119158/1‑301 (MQ=255)`  
                                                                                                                                                                                                                                                                                          `cgccgAGTTGACCCAAAAGAACATGCAGAAGGTCAAAGCGATTGGGTATATCCGAGAGGCTATCGGGGAGAGTATGCTTGAAGGATACTCAATGGACCGCATTAAGCGAGTCACAGAAGGACAGTTCATTCGGGAAGCCAATATGGTAACTGAGAACTATCGCCGTGACTACCAAACAATATTCGCACAGCAACTTGGTGGTACTCAAAGTGCTGCAAGTCAGATTGACGAAATCTATAAGAGGGAACAGAAACAGAAGAGTAAGCTACAGATGGTTCTGGACCCACTGGCTATCATgggg            <  1:7245/301‑1 (MQ=255)`  
                                                                                                                                                                                                                                                                                           `gccgAGTTGACCCCACAGAACATGCAGAAGGTCCAAGCTATTGGGTCTATCCGAGCGGCTATCGGAGAGAGTATGCTTGAAGGTTCCTCAATGGACCGCATTAAGCGAGTCACAGAAGGACAGTTCATTCGGGAAGCCAATATGGTAACTGAGAACTATCGCCGTGACTACCAAGCAATCTTCGCACAGCAACTTGGTGGTACTCAAAGTGCTGCAAGTCAGATTGACGAAATCTATAAGAGCGAACAGAAACAGAAGAGTAAGCTACAGATGGTTCTGGACCCACTGGCTATCATGGGGt           <  1:168875/301‑1 (MQ=255)`  
                                                                                                                                                                                                                                                                                               `aGTTGACCCCACAGAACATGCAGAAGGTCCAAGCTATTGGGTCTATCCGAGCGGCTATCGGAGAGAGTATGCTTGAAGGTTCCTCAATGGACCGCATTAAGCGAGTCACAGAAGGACAGTTCATTCGGGAAGCCAATCTGGTAACTGAGAACTATCGCCGTGACTACCAAGCAATCTTCGCACAGCAACTTGGTGGTACTCAAAGTGCTGCAAGTCAGATTGACGAAATCTATAAGAGCGAACAGAAACAGAAGAGTAAGCTACAGATGGTTCTGGACCCACTGGCTATCATGGGGTCTTc       >  1:138974/1‑301 (MQ=255)`  
                                                                                                                                                                                                                                                                                               `aGTTGACCCCACAGAACATGCAGAAGGTCCAAGCTATTGGGTCTATCCGAGCGGCTATCGGAGAGAGTATGCTTGAAGGTTCCTCAATGGACCGCATTAAGCGAGTCACAGAAGGACAGTTCATTCGGGAAGCCAATATGGTAACTGAGAACTATCGCCGTGACTACCAAGCAATCTTCGCACAGCAACTTGGTGGTACTCAAAGTGCTGCAAGTCAGATTGACGAAATCTATAAGAGCGAACAGAAACAGAAGAGTAAGCTACAGATGGTTCtctgtctcttatacacatctccgagccc       >  1:339323/1‑274 (MQ=255)`  
                                                                                                                                                                                                                                                                                                    `aCCCCACAGAACATGCAGAAGGTCCAAGCTATTGGGTCTATCCGAGCGGCTATCGGAGAGAGTATGCTTGAAGGTTCCTCAATGGACCGCATTAAGCGAGTCACCGAAGGACAGTTCATTCGGGAAGCCAATATGGTAACTGAGAACTATCGCCGTGACTACCAAGCAATCCTCGCACAGCAACTTGGTGGTAATCAAAGTGCTGCAAGTCAGATTGACGAAATCTATAAGAGCGAACAGAAACAGAAGAGTAAGCTACAGATGGTTCTGGACCCACTGGCTATCATGGGGTATTCCGCtt  >  1:102575/1‑300 (MQ=255)`  

                                                                                                                                                                                                                                                                                                       `|`                                                                                                                                                                                                                                                                                                           
`TTCCTCAAGACTATCGGTGCGGTATTCCATGAAGAGTACACACGAGATGGTCAATTTCAGTTATTTACAATCACGAAAGGAGGATAACCATATGTGTTGGGCAGCCGCAATACCTATCGCTATATCTGGCGCTCAGGCTATCAGTGGTCAGAACGCTCAGGCCAAAATGATTGCCGCTCAGACCGCTGCTGGTCGTCGTCAAGCTATGGAAATCATGAGGCAGACGAACATCCAGAATGCTGACCTATCGTTGCAAGCTCGAAGTAAACTTGAGGAAGCGTCCGCCGAGTTGACCTCACAGAACATGCAGAAGGTCCAAGCTATTGGGTCTATCCGAGCGGCTATCGGAGAGAGTATGCTTGAAGGTTCCTCAATGGACCGCATTAAGCGAGTCACAGAAGGACAGTTCATTCGGGAAGCCAATATGGTAACTGAGAACTATCGCCGTGACTACCAAGCAATCTTCGCACAGCAACTTGGTGGTACTCAAAGTGCTGCAAGTCAGATTGACGAAATCTATAAGAGCGAACAGAAACAGAAGAGTAAGCTACAGATGGTTCTGGACCCACTGGCTATCATGGGGTCTTCCGCTG  >  NC_001604.1/27637‑28229`

  

Alignment Legend

Aligned base mismatch/match (shaded by quality score): `ATCG/ATCG` `< 3 ≤` `ATCG/ATCG` `< 11 ≤` `ATCG/ATCG` `< 25 ≤` `ATCG/ATCG` `< 36 ≤` `ATCG/ATCG` `< 38 ≤` `ATCG/ATCG`

Unaligned base: `atcg`    Masked matching base: `atcg`    Alignment gap: `‑`    Deleted base: `‑`
